# Mitochondrial Quality Control in Alzheimer’s Disease: Insights from *Caenorhabditis elegans* Models

**DOI:** 10.3390/antiox13111343

**Published:** 2024-11-01

**Authors:** Upasana Ganguly, Trae Carroll, Keith Nehrke, Gail V. W. Johnson

**Affiliations:** 1 Department of Anesthesiology and Perioperative Medicine, University of Rochester Medical Center (URMC), Rochester, NY 14642, USA; upasana_ganguly@urmc.rochester.edu; 2 Department of Pathology, University of Rochester Medical Center (URMC), Rochester, NY 14642, USA; trae_carroll@urmc.rochester.edu; 3 Department of Medicine, Nephrology Division, University of Rochester Medical Center (URMC), Rochester, NY 14642, USA; keith_nehrke@urmc.rochester.edu

**Keywords:** Alzheimer’s disease, mitochondria, *Caenorhabditis elegans*, model organism, mitochondria quality control, mitophagy, aging, neurodegeneration

## Abstract

Alzheimer’s disease (AD) is a complex neurodegenerative disorder that is classically defined by the extracellular deposition of senile plaques rich in amyloid-beta (Aβ) protein and the intracellular accumulation of neurofibrillary tangles (NFTs) that are rich in aberrantly modified tau protein. In addition to aggregative and proteostatic abnormalities, neurons affected by AD also frequently possess dysfunctional mitochondria and disrupted mitochondrial maintenance, such as the inability to eliminate damaged mitochondria via mitophagy. Decades have been spent interrogating the etiopathogenesis of AD, and contributions from model organism research have aided in developing a more fundamental understanding of molecular dysfunction caused by Aβ and toxic tau aggregates. The soil nematode *C. elegans* is a genetic model organism that has been widely used for interrogating neurodegenerative mechanisms including AD. In this review, we discuss the advantages and limitations of the many *C. elegans* AD models, with a special focus and discussion on how mitochondrial quality control pathways (namely mitophagy) may contribute to AD development. We also summarize evidence on how targeting mitophagy has been therapeutically beneficial in AD. Lastly, we delineate possible mechanisms that can work alone or in concert to ultimately lead to mitophagy impairment in neurons and may contribute to AD etiopathology.

## 1. Introduction

Alzheimer’s disease (AD) is a progressive neurodegenerative disorder and is the leading cause of dementia. As estimated by the Alzheimer’s Association, 6.9 million individuals are living with AD in the United States, which is the fifth leading cause of death in elders above 65 years of age [1]. As life expectancy continues to increase globally, the incidence of AD is also expected to increase, presenting an immense healthcare and economic burden. AD is predominantly characterized by neurodegeneration in the cerebral cortex and hippocampus. Its pathology has classically been defined by the formation of extracellular plaques, consisting of deposits of amyloid beta (Aβ), which is a cleavage product of the amyloid precursor protein (APP), and intraneuronal accumulations of neurofibrillary tangles (NFTs), which predominantly comprise abnormally modified tau [2,3,4,5,6,7,8,9].

AD can be categorized into familial AD (FAD, proven genetic association) and sporadic AD (SAD, uncertain etiology) [10]. Approximately 5–10% of AD cases are caused by heritable, autosomal dominant mutations with an early age of onset (EOAD, <65 years) and are thus classified as FAD. FAD is caused by mutations in the *APP* (21q21. 2–3), presenilin 1 (*PSEN1*, 14q24.2), and presenilin 2 (*PSEN2*, 1q42.13) genes [11]. The remaining 90–95% of AD cases do not possess an identified root cause but rather manifest spontaneously late in life (LOAD, >65 years) and are thus classified as SAD [12]. SAD is a complex and multifactorial disease, and more than twenty demographic, genetic, and environmental risk factors that increase the likelihood of individuals developing SAD have been identified [13]. Several genetic risk factors for SAD have been identified, of which apolipoprotein E (*ApoE*, 19q13.32), particularly the ɛ4 allele of ApoE, significantly increases the risk of developing AD [14]. Because of the number of complex factors that modify AD development and the difficulty associated with studying AD in living humans, research frequently relies on model organisms to interrogate specific molecular mechanisms and test interventions for AD.

Model organisms have played a key role in elucidating the early-disease-relevant changes that occur in AD. Among the common themes that have emerged from decades of research, including work in *C. elegans*, an inability to deal appropriately with mitochondrial dysfunction stands out. Mitochondria are multifaceted organelles that act as bioenergetic reservoirs and information-processing organelles, receiving and transmitting cellular signals that regulate metabolism and energy sensation, among other critical processes [15]. Neurons are energetically demanding cells that rely heavily on mitochondria. Mitochondria contribute to neuronal health through maintaining normal ATP production, membrane potential, oxidative stress responses, axonal transport, and calcium homeostasis [16], and mitochondrial dysfunction can contribute to neurodegeneration through a similar broad range of effectors. As such, therapies that support mitochondrial function are beneficial in AD models [17]. Herein, the insights from *C. elegans* AD models are reviewed, paying particular attention to the diverse ways by which mitochondria may contribute to AD etiology and progression.

In addition to mitochondria contributing to neuronal health, these organelles have evolved mechanisms to self-regulate by responding to stress through a coordinated repertoire of mitochondrial quality control (MQC) mechanisms [18]. Under physiological conditions, MQC is maintained through the careful balance of mitochondrial fission, fusion, and mitochondrial biogenesis and elimination. In times of stress, mitochondria become damaged, and the cell must make a difficult choice: to selectively degrade potentially harmful mitochondria through autophagic machinery—a process called mitophagy—or to upregulate stress response proteins to promote mitochondrial survival and energy production with the remaining mitochondria—a process known as the mitochondrial unfolded protein response (mito-UPR). *C. elegans* has provided a tractable model for interrogating these complex adaptive responses, and considering how the chronic activation of compensatory regimens may become maladaptive over time or with age is a fundamental concern in neurodegenerative disease.

## 2. *C. elegans* as a Model Organism to Study Neurodegeneration and Mitochondrial Biology in Alzheimer’s Disease (AD)

*C. elegans* was first introduced as a model organism for molecular and developmental biology by Sydney Brenner in 1974 [19]. Since then, the ranks of worm aficionados have risen to include thousands of laboratories worldwide. *C. elegans* possess select advantages that make them ideal model organisms for studying the fundamental cell biology of human disease. An invariant body plan consists of 959 somatic nuclei in hermaphrodites and 1031 in males. Having two sexes facilitates conventional genetic approaches. Worms develop from embryos to adults in ~3 days, and with a lifespan of ~two weeks, they are a premier model for studying aging. Other notable features include ease of maintenance, a short lifecycle, robust, easily discernible, and well-annotated phenotypes, and, perhaps most importantly, when considering them as a model for human disease, a conserved cellular and molecular architecture [20,21]. They possess a recognizable repertoire of tissue types, such as the intestinal, neuronal, muscular, and hypodermal types, communicating and interacting in familiar ways. Moreover, their transparent bodies permit non-invasive visualization modalities such as fluorescent biosensors, enabling studies at the single-cell level, often in live-behaving animals. Approximately 42% of genes responsible for various human diseases also have clear orthologs in worms, making them an invaluable choice for studying disease-associated molecular pathogenesis and therapeutics [22]. Extensive knowledge of these animals is cataloged in publicly available resources such as Wormbase (http://www.wormbase.org/ (accessed on 11 September 2024)), and the *Caenorhabditis* Genetics Center (https://cgc.umn.edu/ (accessed on 11 September 2024)) houses and disseminates many of the published strains.

The *C. elegans* nervous system consists of 302 neurons with a well-annotated neural connectome and possesses many functional similarities with higher organisms, including canonical neurotransmitters (γ-aminobutyric acid, dopamine, serotonin, acetylcholine), their receptors, and neuromodulators [23]. Due to the worm’s relative anatomical simplicity and limited repertoire of behaviors, correlating neuronal activity with cognate behavioral outputs has provided an easy way to assess how neurons and circuits integrate information to inform downstream processes. Ample forward genetic screens and the pioneering use of optogenetic approaches to modulate neuronal function in worms provide novel ways to interrogate the molecular foundations of neurobiology. *C. elegans* also possesses a remarkable conservation of mitochondrial biology which makes it an ideal model system for investigating complex processes that are difficult to study in higher organisms [24]. For example, *C. elegans* has been utilized as an in vivo model to study mitochondrial dynamics [25,26]. Many genetic knockouts of various factors controlling mitochondrial dynamics, signaling, and quality control mechanisms are embryonic lethal in mice, but the loss of orthologous genes in *C. elegans* is generally tolerated, facilitating genetic approaches to study mitochondrial input into disease [24].

**Figure 1:**
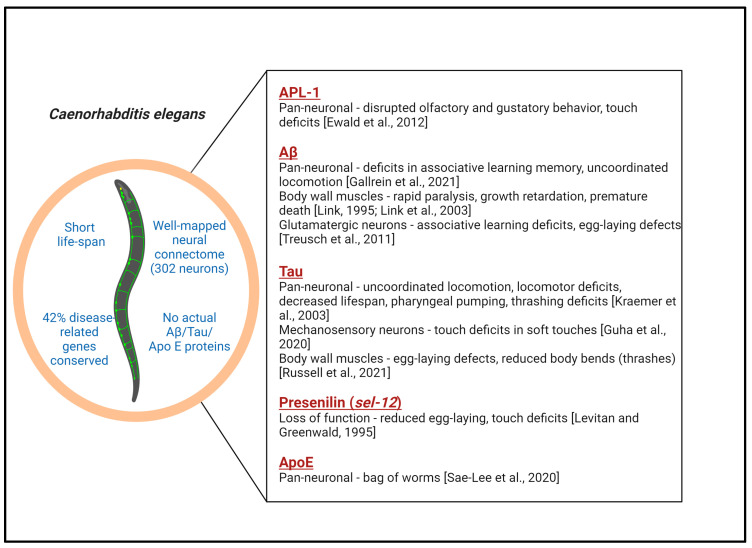
Behavioral phenotypes observed in *Caenorhabditis elegans* models of Alzheimer’s disease. Summarized evidence of behavioral changes observed in worms expressing APL-1, Aβ, tau, presenilin (sel-12), and apolipoprotein E (ApoE) pan-neuronally or in specific neuronal subsets (glutamatergic or mechanosensory) or body wall muscles [30,31,32,33,34,35,36,37,38,39]. Image created using https://www.biorender.com.

One of the most extensively used approaches for investigating specific neurodegenerative diseases in *C. elegans* involves either manipulating the expression level of an orthologous gene known to impact human disease or alternatively introducing human disease-related genes, often containing mutations that impact disease severity, to produce disease-related phenotypes in transgenic worms [27]. While individual worm models of AD are described in detail below and in Figure 1, it is worth mentioning here a study by Apostolakou et al. that utilized protein–protein interaction (PPI) networks to model amyloid precursor protein (APP) and tau in humans and their orthologs APL-1 (APP-related protein 1) and PTL-1 (protein with tau-like repeats-1) in worms [28]. Using global network alignment, this in silico study suggested a high degree of conservation between *C. elegans* and humans, albeit with some important limitations (as discussed in the next section and in Alvarez et al. [29]). Some of the limitations described below can even be perceived as advantages when introducing AD-associated pathologic human proteins through transgenic approaches, allowing for reductionist models to identify defects in highly conserved processes. Fewer neurons mean more direct genotype–phenotype relationships and simply behavioral correlates of dysfunction. The intricate characterization of individual neurons within the model and how they relate to one another allows for a more granular interrogation at a level that is not currently achievable in mammals. A shorter lifespan allows for a rapid assessment of how age impacts “disease” outcomes. With this in mind, AD models in worms can be classified into APL-1 models, Aβ models, PTL-1 models, tau models, apolipoprotein E models, and presenilin models, as follows (Figure 1).

### 2.1. APL-1 Models

Worms possess a single APP-related gene, *apl-1*. Like human APP, APL-1 contains an extracellular domain, a transmembrane domain, and a cytosolic domain, but it does not contain an Aβ domain [40]. In addition, there are no β-secretase enzymes that would cleave Aβ from the precursor protein. However, α-secretases (SUP17, ADM4) are known to cleave APL-1 into a large extracellular domain (sAPL-1) and a small cytoplasmic domain [41]. Hornsten et al. used transcriptional and translational GFP reporter transgenes to map APL-1 expression in different worm tissues [42]. APL-1 was expressed in neurons, muscles, the hypodermis, and supporting cells in adult animals. Nevertheless, information on the role of APL-1 in worms and its association with AD pathology is extremely limited. Several studies have utilized *C. elegans* strains with *apl-1* loss of function or overexpression to map expression patterns and phenotypic effects. Perhaps the most interesting of these studies reported that pan-neuronal *apl-1* expression disrupted olfactory and gustatory learning behavior and touch habituation in transgenic worms compared to the wild type [30]. This work also showed that APL-1 signaling required the activity of DAF-16/FOXO transcription factor and DAF-12 (nuclear hormone receptor), which modulated the metabolic cascades controlling development, body size, and egg-laying in worms [30], and subsequent work demonstrated negative regulation by the insulin/insulin-like signaling (IIS) receptor DAF-2 acting upstream of DAF-16 [40]. Despite these findings, the significance of APL-1 phenotypes to the pathology of AD is still uncertain. However, the connection between APP and insulin pathways first established in worms was later investigated using in vitro (primary cortical neurons) and in vivo (mouse) AD models where insulin and insulin growth factor-I (IGF-I) were shown to promote the dephosphorylation of APP and non-amyloidogenic processing [43]. Moreover, insulin resistance is a feature of metabolic syndrome and type 2 diabetes, which are critical risk factors for AD [44,45,46].

### 2.2. Amyloid-β Models

The first transgenic *C. elegans* expressing human Aβ was developed by Christopher Link in 1995 [31]. These animals exhibited a muscle-specific accumulation of Aβ (as detected by thioflavin-S staining and anti-Aβ reactivity), leading to age-dependent progressive paralysis. The coexpression of transthyretin in the transgenic worms reduced Aβ deposits [31]. Fay et al. demonstrated that L17P and M35C mutations in Aβ transgenic worms completely abolished the formation of thioflavin-S-reactive deposits, implicating the necessity of these two amino acid residues for in vivo amyloid deposits [47]. Proteomic analysis identified six chaperone proteins that bound Aβ, including HSP-16—which co-localized with intracellular Aβ and, when overexpressed, provided protection against Aβ toxicity, highlighting the role of chaperones in Aβ metabolism [48,49].

A key question about AD pathology is whether late-onset toxic protein aggregates are formed in a stochastic process or whether aging causes constitutively aggregating proteins to become toxic by interfering with clearance pathways. To understand this dilemma, Cohen et al. targeted the IIS pathway that regulates stress resistance in worms via its receptor DAF-2 [50]. As discussed in the above section, DAF-2 negatively regulates DAF-16 to control entry into a stress-resistant alternative larval stage termed “dauer”, contributing to stress resistance in non-dauer worms and modulating lifespan [51,52]. Hence, *daf-2* mutant worms exhibit resistance to a variety of stressors and live longer [53,54,55,56]. Cohen et al. showed that *daf-2* RNAi decreased Aβ1-42-mediated paralysis in aged worms and suggested that this result supported the direct effect of aging on toxicity rather than a stochastic process. Moreover, they demonstrated the need for transcription factors heat shock factor-1 (HSF-1) and DAF-16 acting downstream of DAF-2 to modulate pathologic outcomes [50].

Cohen’s study further asked whether Aβ proteotoxicity was mediated by high-molecular-weight aggregates by measuring their abundance following *daf-2*, *daf-16*, or *hsf-1* RNA interference (RNAi). While *hsf-1* RNAi animals had the most aggregates, *daf-2* RNAi animals had more aggregates than *daf-16* RNAi animals but were relatively protected [50]. These findings are inconsistent with high-MW aggregate abundance being the sole arbiter of toxicity, but it is also likely that other functions of IIS contribute to pathologic outcomes. These data supported the fact that insulin resistance is an important risk factor in AD.

Drugs like metformin, which are the primary therapy for type 2 diabetes mellitus (T2DM), delayed rapid paralysis in worms expressing Aβ in muscles [57]. Caloric restriction also reduced Aβ toxicity in worm models in an HSF-dependent pathway, causing reduced proteotoxicity and enhanced thermotolerance and lifespan [58]. Similar findings were recapitulated in AD mouse models where caloric restriction attenuated Aβ neuropathology [59,60,61,62]. In a more recent study, long-term caloric restriction proved to be neuroprotective in AD transgenic mice, leading to improved glucose metabolism, increased neuronal integrity and activity, reduced cognitive decline, and increased autophagy [63].

Further work from the Link lab resulted in a transgenic model with a temperature-dependent induction of Aβ in muscles. These transgenic animals were normally propagated at 16 °C but at 23 °C exhibited a 4–5-fold increase in Aβ mRNA and protein levels accompanied by rapid paralysis [32]. Gene expression analysis identified 67 upregulated and 240 downregulated genes, with genes coding for A-B-crystallin (CRYAB) and tumor necrosis factor-induced protein 1 (TNFAIP1) upregulated both in the transgenic worm model as well as post-mortem human AD brain tissue [32].

Although these transgenic worm models provided a plethora of information on the pathophysiological roles of Aβ, they were restricted to expressing Aβ in muscle tissues. In 2011, another transgenic *C. elegans* model was developed that expressed human Aβ1-42 in glutamatergic neurons (a subset of neurons vulnerable in AD) using the eat-4 promoter [38]. These worms demonstrated age-dependent neurodegeneration: 48% and 25% of the worms had intact glutamatergic neurons at day three and day seven, respectively; these transgenic worms also established a functional association of Aβ toxicity and endocytic trafficking pathways. Gallrein et al. developed transgenic animals with the fluorescent-sub-stoichiometric labeling of Aβ1-42, enabling the in vivo tracking of the peptide [33]. Their study observed the selectivity of Aβ aggregation—the pathology initiated in a subset of neurons in the anterior-head ganglion (six IL2 neurons). RNAi-mediated protein depletion in these neuronal subsets could systemically halt protein aggregation and pathology.

### 2.3. PTL-1 Models

Tau is a microtubule-binding protein that supports the neuronal cytoskeleton [64]. The aggregation of tau protein, particularly that which is heavily post-translationally modified, is an important contributor to many neurodegenerative diseases, including AD, Frontotemporal dementia (FTLD), Progressive supranuclear palsy (PSP), Pick’s Disease, and Parkinson’s disease. The initial notion in the field was that tau aggregates form neurofibrillary tangles (NFTs) that impair synaptic communication between neurons, resulting in neurodegeneration [9]. However, studies over the past 20 years have shown that NFTs are not the primary toxic species and that in AD models (mouse or *Drosophila*), when soluble tau is decreased, NFTs continue to increase with improved behavior and the halting of neuronal loss [65,66,67,68]. The *C. elegans* genome encodes a protein with tau-like repeats (*ptl-1*) that bears 50% homology with human tau [69]. While it is the sole nematode microtubule-associated protein (MAP) protein, there are three family members in mammals—MAPT (tau), MAP2, and MAP4. PTL-1 is widely expressed in many neurons, including mechanosensory neurons [69,70,71,72]. Like human tau, PTL-1 is also a microtubule-binding protein with multiple potential phosphorylation sites and exists in two isoforms (PTL-1A and PTL-1B) of 413 and 453 amino acids with four or five tandem repeats [69]. *Ptl-1* loss of function impaired touch sensitivity; decreased lifespan; resulted in abnormal morphology in mechanosensory and GABAergic neurons, which parallels some features seen in AD pathology; sensitized neurons to damage from mechanical stress; and altered stress tolerance and aging [73,74,75]. However, AD is not associated with tau loss of function but rather misfunction, PTL-1 is not known to aggregate, and the recent humanization of ptl-1 with human MAPT and disease-associated variants failed to elicit a notable phenotype [76]. Finally, human tau expression in these worms cannot robustly rescue the loss of *ptl-1*, indicating that PTL-1 and human tau are functionally divergent [73].

### 2.4. Tau Models

Several *C. elegans* models that overexpress human tau in wild-type or mutant forms have been developed, with the latter exhibiting greater toxicity [34,77,78,79]. Early studies found that the pan-neuronal overexpression of human tau led to progressive uncoordinated locomotion, insoluble tau accumulation, and neurodegenerative phenotypes consisting of bulges and gaps in nerve cords followed by neuronal loss, while a mutant form of tau (Frontotemporal dementia with parkinsonism chromosome 17 type (FTDP-17) tau (P301L, V337M)) demonstrated a more severe phenotype [34]. A similar approach by Brandt et al. compared the pan-neuronal overexpression of wild-type human tau and pseudohyperphosphorylated (PHP) tau [78]. This study was crucial as it demonstrated that human tau could be phosphorylated in *C. elegans* and exhibited conformational changes like PHP tau found in human AD. Another study showed that the pan-neuronal expression of A152T*MAPT* phenocopied defects was associated with AD pathology—locomotory defects, decreased lifespan, age-dependent changes in neuronal morphology, and GABAergic neurodegeneration [77]. A subsequent study by Butler et al. showed that the pan-neuronal overexpression of A152T tau (1N4R) exhibited impaired thrashing behavior, reduced pharyngeal pumping, decreased associative learning behavior, and an impaired hatching and development of worms [79]. An interesting finding of this study was that the overexpression of A152T tau impacted the axonal transport of synaptic vesicles which showed that tau phosphorylation influenced microtubule-based cargo transport. While it was informative and pioneering, as expanded on below, the overexpression of wild-type tau was not benign in these models, begging the question of whether reducing expression levels would result in a selective deficit in lines expressing mutant, disease-related tau.

Our lab addressed this question using a single-copy gene insertion approach comparing wild-type tau to a phosphomimetic mutation of T231 to E mimicking the phosphorylation of an epitope that appears early in AD pathology or an acetylmimetic mutation of K274/K281 to Q that mimics an epitope found later in the disease, with the thought that this approach would allow us to identify the earliest molecular phenotypes associated with dysfunction [35]. Though subtle, there was a discrete and select deficit in the behavioral response to light touch in worms expressing mutant tau in touch neurons compared to wild-type and a mitochondrial phenotype described in more detail below [35]. Recent work from the Kraemer group also showed that tau expression levels greatly impact phenotypes, with single-copy strains being useful for identifying enhancers of toxicity [80].

Returning to overexpression models, Kraemer et al. used RNAi screening to identify genes that enhance the tau-induced uncoordinated phenotype in worms [81]. A total of 75 targets were identified, of which 15 were non-specific to tau and enhanced the locomotory phenotype in non-tau Unc strains (*unc-3*, *unc-76*, *unc-78*, *unc-87*). Of the 60 that were tau-specific, 38 had human counterparts, with 6 being previously implicated in tau pathology in human or animal tauopathy models [81]. Directly correlating the action of these enhancers to an effect on tau was difficult, however, given that many of them had broad regulatory roles in cell biology, and several of the target genes had been shown to phosphorylate tau itself, which was counterintuitive given that hyperphosphorylated tau is associated with disease. It was suggested that decreased phosphorylation in this model might lead to less aggregation and more soluble tau, which could be the toxic moiety.

Proteotoxicity is a challenge in neurodegenerative disorders because of the systemic failure to clear aggregated proteins, often more pronounced with aging. The age-dependent accumulation of toxic aggregates of tau often poses a challenge to the endoplasmic reticulum unfolded protein response (UPR^ER^) in the aging brain, resulting in ER stress. As we discussed, Kraemer et al. identified several modifiers of tau toxicity [81]. A further study by the Kraemer group utilized genome-wide RNAi screening in a worm model overexpressing human tau pan-neuronally and identified that UPR^ER^ is critical for tau proteostasis [82]. The loss of function of UPR^ER^ master transcriptional regulator *xbp-1* (X-box binding protein 1) and *atf-6* (Activating transcription factor 6) exacerbated tau toxicity, whereas the overexpression of *xbp-1* ameliorated tau-induced phenotypes. This corroborates an earlier study that showed that polymorphism in the XBP-1 promoter in humans was a risk factor for AD in a Chinese Han population [83]. These studies emphasize the fact that maladaptive ER stress is an important feature of AD pathology, the details of which were discussed in some excellent reviews [84,85].

Genetic screens performed by the Kraemer group in their overexpression model identified the suppressors of tauopathy genes *sut-1* and *sut-2* as suppressors of the tau phenotype [86,87]. The SUT-1 protein physically interacts with UNC-34 which is an important mediator of signaling pathways in actin dynamics, particularly axonal pathfinding and neuronal migration during worm development [87]. The SUT-2 protein binds *C. elegans* HOOK protein ZYG-12, which functions in conserved signaling pathways executing centrosome function and the aggresome-mediated processing of protein aggregates [86]. Thus, the loss of function of *sut-1* or *sut-2* can interfere with axonal dynamics and prevent tau accumulation and neurotoxicity. While there are no human homologs of SUT-1 or SUT-2, these studies were critical as they showed that the loss of a single gene eliminated the toxic effects of human tau in transgenic worms [36].

Dysfunctional mitochondria and tau-mediated neurodegeneration are tightly linked, but the chronology is still debated. Studies from our lab using a single-copy model that lacks apparent tau aggregation revealed that phosphomimetic tau and non-wild-type human tau selectively inhibited oxidative stress-induced mitophagy and reduced mitolysosomal trafficking starting from early adulthood in worms [35]. Mitophagy deficits exhibited by these worms were dependent on the PINK-1 pathway (a critical mediator of mitophagy) but not on DRP-1 (a critical regulator of mitochondrial fission), thereby highlighting the fact that adaptive mitophagy may be a potential target of toxic tau species in AD pathology [88]. A separate model with the pan-neuronal expression of low levels of human tau demonstrated that mitochondrial dysfunctions occur in young animals before tau aggregates begin to form in neurons [89]. These transgenic worms displayed mitochondrial impairment and pronounced locomotor deficits. Although these worms did not accumulate tau in the L1-L4 larval stages, they exhibited reduced body bends compared to wild-type worms and decreased mitochondrial density in ventral and dorsal cord neurons. It was suggested that increased mitochondrial damage and impaired energy generation in these worms drove abnormal locomotion and neuronal dysfunction [89], establishing the role of mitochondrial dysfunction as an early pathological feature of tauopathy.

### 2.5. APOE Models

Human ApoE is important for the metabolism and transport of fat, lipids, and cholesterol in blood and lymph [90]. In the nervous system, ApoE transports cholesterol to neurons via astrocytes [91]. Variation in the gene encoding ApoE is the most predictive risk factor for LOAD [92]. ApoE is a polymorphic protein with three major isoforms, ApoE2, ApoE3, and ApoE4, that differ by single amino acid substitutions [90]. The ApoEε4 and ApoEε2 alleles have opposing effects: ApoEε2 is associated with a reduced risk of AD, whereas ApoEε4 is associated with an increased risk for AD [93]. The mechanism by which this occurs is not clearly defined but hints at the involvement of altered glucose and lipid metabolism. Transgenic *C. elegans* models have been developed to study the effects of ApoE alleles with or without the presence of Aβ. The coexpression of human ApoEε2 with Aβ in worms attenuated amyloid-induced neurodegeneration; ApoEε4 had no neuroprotective ability, and ApoEε3 displayed an intermediate effect and offered neuroprotection only in older animals [94]. However, when ApoE alleles were expressed in the neurons of worms without Aβ, there was no difference in survival compared to wild-type animals. The coexpression of ApoE and APP in worms showed that ApoEε4 acts with APP to exacerbate cholinergic neurodegeneration in AD pathology [39]. Finally, recent work has demonstrated that the VHL-HIF axis is a potent modifier of mortality in transgenic worms expressing toxic ApoE [95], highlighting a novel way of mitigating adverse outcomes. These studies hint at the utility of ApoE worm models in determining the selectivity of neurodegeneration observed in AD.

In addition to AD, ApoE variants have been linked to Parkinson’s disease (PD), depicting a complex relationship between the different PD subtypes and symptoms. An interesting study by Koks et al. identified that different ApoE haplotypes regulate the function of the mitochondrial protein TOMM40, thereby establishing a link between mitochondria and ApoE [96]. A similar association has been observed in AD patients [97]. However, the causal relationship between ApoE and TOMM40 in the pathogenesis of AD has not been studied in *C. elegans* yet.

### 2.6. Presenilin Models

Autosomal dominant familial Alzheimer’s disease (FAD) is caused by mutations in three genes—*APP*, *Presenilin-1 (PSEN1)*, and *Presenilin-2 (PSEN2)*. Mutations in the *PSEN1* gene are the most common cause of FAD, followed by variants in APP, whereas fewer variants in *PSEN2* lead to AD [98,99,100,101]. Presenilin-1 acts as the catalytic subunit of the γ-secretase enzyme complex (GSEC), consisting of transmembranous proteases that cleave proteins like APP to generate Aβ of varying lengths (37–43 amino acids) [101,102]. However, how PSEN1 mutations lead to disease pathology in FAD is still debatable. On one hand, a group of researchers showed that *PSEN1* mutations result in the pathological gain of function in the endoproteolytic processing of APP, subsequently increasing the production of Aβ42 [103,104]. However, studies on 138 pathogenic mutations in human *PSEN1* revealed that 90% of them reduced Aβ42 and Aβ40 production, whereas 10% of mutations were associated with reduced Aβ42/Aβ40 ratios [105] which indicated that the role of presenilin as part of the GSEC-mediating endolytic processing of APP is limited. Another group of researchers focused on the more crucial intracellular functions of presenilin in learning, memory, and neuronal survival during aging, the loss of which can lead to neurodegeneration and dementia in FAD [106,107].

*C. elegans* has three presenilin genes—*sel-12*, *hop-1*, and *spe-4*. *Spe-4* expression is limited to the male germline [108]. *Sel-12* and *hop-1* (homolog of presenilin) encode proteins that bear 50% and 33% homology to mammalian presenilins, respectively [37,109]. *Sel-12* is expressed in neural and non-neural cells throughout development in worms [110]. A reduction in or complete loss of *sel-12* results in an egg-laying defective (Egl) phenotype in worms, and SEL-12 facilitates LIN-12/Notch signaling [37], much like how PSEN1 functionally supports the endoproteolysis of Notch in mammals and *Drosophila* [111,112,113]. Human presenilins can rescue the egg-laying defects in *sel-12(ar131)* hermaphrodites, while AD-related PSEN mutants cannot, suggesting the conservation of the protein’s function across species [110]. The similarity between SEL-12 and human presenilins suggests that SEL-12 could be the worm PSEN1/2 equivalent relevant to AD.

Because worm APL-1 lacks an Aβ domain, investigators have suggested that select neuronal defects in *sel-12* mutants are caused by facilitated Notch signaling [114], and this is almost certainly true for egg-laying defects [115]. However, many studies have suggested a critical γ-secretase-independent role, which has been difficult to disentangle from the more obvious roles in Notch signaling and APP processing. For example, early work from the Ishii lab demonstrated that *sel-12* mutants demonstrated resistance to oxidative stress and prevented mitochondrial dysfunction-induced apoptosis through a process that was related to abnormal calcium release from the endoplasmic reticulum [116]. The Norman lab subsequently expanded on these findings to show that mitochondrial dysfunction occurs via defects in calcium homeostasis, independent of Notch signaling, γ-secretase proteolytic activity, and amyloid plaque formation [117]. *sel-12* mutants have elevated ER–mitochondria calcium signaling, leading to oxidative stress and neurodegeneration, recapitulating AD pathology [118]. Presenilin loss of function also causes neurodegeneration in worms by the hyperactivation of the mTORC1 signaling cascade, which causes the dysregulation of proteostasis and autophagy in these worms that can be rescued by the genetic and pharmacologic inhibition of the mTORC1 pathway [119]. Presenilin loss increases mitochondrial ROS generation and activates the mTORC1 pathway by suppressing the SKN-1/Nrf antioxidant pathway in neurons [120]. These studies point to the critical involvement of mitochondria and mTORC1 pathways in neurodegeneration, pointing to new therapeutic avenues in the treatment of AD.

### 2.7. The Limitations of the C. elegans Model for Studying AD Pathology

There are certain limitations of utilizing *C. elegans* as models to study AD pathology that are also worth mentioning. A lifespan measured in weeks may be an experimental advantage, but given that AD is a disease of age and takes years to fully manifest in affected individuals, it is unclear that the time scales allow for appropriate temporal referencing. Another caveat is that neuronal processes are generally short, and since the worm genome lacks the coding potential for a voltage-gated sodium channel, neurons generally operate through calcium-mediated depolarization rather than action potentials [121,122]. And of course, with only 302 neurons, higher-order tissue structures like the cerebral cortex or hippocampus do not exist. Similarly, without a dedicated vasculature or immune system, certain aspects of AD are impossible to account for, such as the contribution of neuroinflammation.

Worms also lack some of the risk factors associated with AD. For example, worms do not possess ApoE [29], the e4 form of which is one of the most common risk factors for LOAD. Moreover, while there are APP- and tau-like proteins in worms, it is unclear whether these proteins are truly orthologs in the functional sense and whether they contribute to AD-like phenotypes upon dysfunction—caveats that are explored in depth below.

So, what does this say about the worm model? Our take is that the general conservation of the genomic coding potential and protein interaction network is reassuring, in that the molecules and processes through which AD-related protein phenotypes might occur are likely to perform similar functions in worms and mammals, albeit requiring verification, but that conclusions based on endogenous APL-1 and PTL-1 may be more tenuous. In line with this conclusion, a more common approach over the past several decades has been to express human Aβ or tau exogenously.

## 3. Mitophagy in Alzheimer’s Disease

Autophagy maintains cellular homeostasis by degrading parts of the cytoplasm, organelles, proteins, lipids, and nucleic acids [123]. There are three forms of autophagy, macroautophagy, chaperone-mediated autophagy (CMA), and microautophagy, the mechanisms of which have been detailed in some excellent reviews [124,125,126]. Based on specificity, macroautophagy (or autophagy for short) can be classified by selectivity. Bulk autophagy is the indiscriminate degradation of cytoplasmic components in the face of starvation. Selective autophagy is more discrete in the choice of ‘cargo’ for degradation, often including organelles like mitochondria (mitophagy), the endoplasmic reticulum (ERphagy), lysosomes (lysophagy), peroxisomes (peroxophagy), ribosomes (ribophagy) or macromolecular complexes like protein or RNA aggregates (aggrephagy), lipids (lipophagy), or intracellular pathogens (xenophagy) [123]. Selective autophagy requires the recognition of the specific cargo which is often mediated by cargo ubiquitylation and the involvement of specific autophagy receptors (p62/SQSTM1, NDP52/CALCOCO2, TAX1BP1, NBR1, optineurin). These key players contain an LC3-interacting domain (LIR) and ubiquitin-binding domain that act as a bridge to facilitate the binding of cargo [127,128,129]. Despite the differences in cargo selection, the two types of autophagy share degradation machinery—the formation of a phagophore or isolation membrane that engulfs the cargo to form double membrane-bound vesicles called autophagosomes (APs) which fuse with lysosomes to form autolysosomes (ALs) where the cargo is degraded by lysosomal proteases. An interesting observation in this regard is that mitochondria, which are degraded by selective autophagy, nevertheless serve as important sources for the autophagosomal membrane for bulk autophagy [130]. During nutrient starvation (major trigger for bulk autophagy), the phosphatidyl ethanolamine (PE) synthesized in mitochondria acts as the only source of PE utilized to covalently attach autophagy-regulating proteins (ATGs) to the autophagosomal membrane [131].

The mitochondrial network is dynamic and is monitored by several MQC pathways including mitophagy, whose molecular components are fundamentally conserved from yeast to mammals [132]. Several excellent reviews have summarized mitophagy defects in AD brains and different AD models [133,134,135]. One of the prominent features of AD is the accumulation of dysfunctional mitochondria and autophagic vacuoles (AVs) in different neuronal sub-compartments (soma, axons, synapses) and degenerating neurites [136,137]. Deficits in synaptosomal mitophagy lead to a reduced clearance of Aβ plaques and cause the accumulation of Aβ in AD [138,139]. Transgenic mice expressing full-length wild-type human tau possess mitophagy deficits, as indicated by the increased levels of mitophagy markers COX IV and TOMM20 and the altered mtDNA–genomic DNA ratio [140]. These studies also demonstrated that tau is critical in mitophagy dysregulation in AD. Tau accumulation causes the direct insertion of the protein into the mitochondrial membrane, leading to mitochondrial hyperpolarization and defects in PINK1/Parkin recruitment [138]. A decreased expression of the proteins that play integral roles in autophagy and mitophagy machinery has been observed in fibroblasts from AD brains, such as ULK1, AMBRA1, BNIP3, NIX/BNIP3L, FUNDC1, VDAC1, VCP/P97, OPTN, ATG5, ATG12, and Beclin-1 [141]. Post-mortem studies in the hippocampus from AD patients demonstrate 30–50% reduced mitophagy compared to age-matched controls—mitophagy was determined by electron microscopy (EM) by the co-localization of mitochondrial TOMM20 with lysosome-associated membrane glycoprotein 2 (LAMP2) [139]. In addition to reduced mitophagy, mitochondrial structural deformities were observed in these hippocampal neurons together with reduced LC3 recruitment, increased AMPK phosphorylation, deficits in AMPK binding target proteins (ULK1, TBK1), and defects in mitophagosome–lysosome fusion [139]. The expression of Swedish mutations in APP can cause mitophagy dysregulation and mitochondrial structural defects in mAPP-HT22 cells [142]. This study observed increased mRNA and protein levels of mitochondrial fission genes (Drp1, Fis1) and decreased levels of mitochondrial fusion genes (*Mfn1*, *Mfn2*, *Opa1*), mitochondrial biogenesis genes (*PGC1α*, *NRF1*, *NRF2*, *TFAM*), mitophagy genes (*PINK1*, *BNIP3L*), and autophagy genes (*ATG5*, *LC3BI*, and *LC3BII*). Fibroblasts from FAD with PSEN1 mutations report lysosomal deficits, lysosomal alkalization, and decreased cathepsin D activity because of defects in vATPase targeting lysosomes [143]. Transcriptomic analyses of post-mortem AD brains reveal that ApoE4 carriers demonstrate an upregulated gene expression of LC3, p62/SQSTM1, NBR1, OPTN, and BNIP3 [144]. These pieces of evidence point to the direct involvement of dysregulated mitophagy in AD pathogenesis; however, whether this is a cause or consequence of AD pathology is still debated.

**Figure 2:**
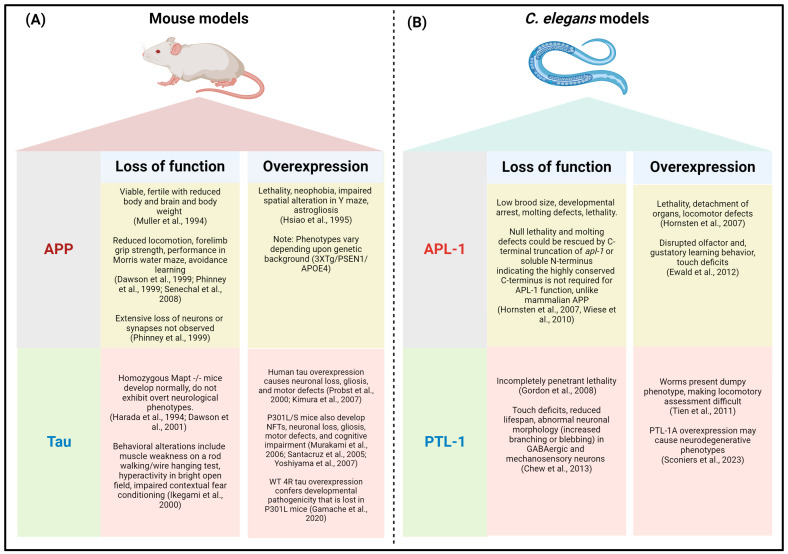
Comparative analyses of phenotypes in mouse models and *Caenorhabditis elegans* models of AD. (**A**) Overview presenting different behavioral observations owing to loss of function or overexpression of APP and tau in mice. While APP-null mice exhibit reduced viability and many behavioral phenotypes, tau-null mice develop normally with subtle behavioral alterations [145,146,147,148,149,150,151]. Overexpression of APP or tau leads to multitude of behavioral impairments, including lethality in APP-overexpressing mice [152,153,154,155,156,157,158]. (**B**) Summarizing phenotypic alterations in *C. elegans* with loss of function or overexpression of APL-1 and PTL-1. Null mutations in APL-1 and PTL-1 are lethal (incompletely penetrant in *ptl-1*-null worms) [42,70,73,159]. Overexpression of APL-1 and PTL-1 in worms also leads to presentation of many overt neurological phenotypes [30,42,72,160]. Image created using https://www.biorender.com.

Therefore, employing a reductionist approach in a genetic model organism like *C. elegans* that lacks endogenous Aβ or tau might provide a better insight into the pathology of AD. Of note, the loss of function and overexpression of endogenous APP and tau orthologs give rise to similar phenotypes in mammals and worms, highlighting the likely conservation of key pathways involved in AD between organisms (Figure 2).

Here, we delve into the mechanisms of mitophagy regulation in worms, how worm models contributed to understanding the pathophysiology of mitophagy in AD, and the potential causes of mitophagy dysfunction in AD with an emphasis on knowledge derived from studies in worms. Again, we reiterate the caveat that worms are a model, and there are potential differences between AD models and actual disease pathology. Nevertheless, the use of model organisms aids in discerning cause from correlation by allowing for approaches that are not feasible (nor ethical) in humans and have the potential to drive advances in the field but nevertheless require validation in de facto disease.

## 4. Mitophagy in Worms: Mechanisms and Regulation

Based on its physiological role, mitophagy can be categorized into basal mitophagy, programmed mitophagy, and stress-induced mitophagy [161]. Basal mitophagy is the selective autophagy of mitochondria under steady-state conditions without external stimuli. The level of basal mitophagy varies within tissues and even cell types within the same tissue. Basal mitophagy levels are very low in *C. elegans*, which often necessitates the application of external stress to stimulate mitophagy for experimentation. This form of mitophagy is stress-induced mitophagy, which describes the potent upregulation of mitophagy in response to various exogenous stressors such as nutrient deprivation, exercise, and hypoxia [161,162]. Lastly, certain cell types undergo mitochondrial clearance during their development, a process referred to as programmed mitophagy. A classic example of programmed mitophagy is the elimination of sperm-derived mitochondria from fertilized oocytes in worms [163]. At its core, mitophagy acts as an essential metabolic switch to maintain the health of the mitochondrial network through selective degradation [164]. *C. elegans* are robust model organisms for studying mitophagy in part due to the conservation of many molecular pathways and the availability of several fluorescent mitophagy reporters, which is discussed in detail in Table 1.

Besides mitophagy, worms also possess alternative ways to eliminate or adapt to damaged mitochondria. For example, mitochondria can be eliminated by the formation of exophers—extracellular vesicles that also contain damaged proteins [175]. Neurons with proteotoxic burdens generated and secreted exophers (~4 μm size) that relieved them from stress and enabled them to function better. Subsequent studies by Turek et al. showed that nearly 12% of exophers generated from *C. elegans* body wall muscles contain intact mitochondria [176].

Mitophagy pathways are tightly regulated and involve the convergence of multiple signaling events. Mitophagy induction requires mitochondria to be marked explicitly for mitophagy (depending upon damage, stress, or cellular stimuli). This ‘priming’ of mitochondria also involves the isolation of mitochondria from the mitochondrial network and the involvement of autophagy machinery to detect “eat me” signals on the mitochondria [164]. While mitophagy requires critical components of the autophagy pathway, distinct mitochondrial proteins are involved in mediating the process. Canonical mitophagy pathways can be broadly classified into PINK1/Parkin-mediated mitophagy (ubiquitin-dependent), receptor-mediated mitophagy (ubiquitin-independent), and lipid-mediated mitophagy. Here, we categorized mitophagy into PINK1/Parkin-dependent and -independent pathways, highlighting the information available from studies in worm models.

### 4.1. PINK-1-PDR-1-Dependent Mitophagy Pathway

Mutations in phosphatase and tensin homolog (PTEN)-induced putative kinase-1 (PINK1) and E3 ubiquitin ligase Parkin are the most common cause of autosomal recessive Parkinson’s disease (PD) with early disease onset [177]. PINK-1 acts as a surveillance mechanism or damage sensor for mitochondrial fitness. In healthy mitochondria, PINK-1 is recruited to the inner mitochondrial membrane (IMM) by the mitochondrial TOM and TIM complex and becomes degraded by the action of mitochondrial proteases. Loss in mitochondrial integrity anchors PINK-1 on the outer mitochondrial membrane (OMM). PINK-1 undergoes autophosphorylation and phosphorylates ubiquitin chains (Ub) or poly-ubiquitin (Poly-Ub) on other OMM proteins at Ser-65 [132]. This event serves as a signal for both the recruitment of PDR-1 from the cytosol to the damaged mitochondria and the phosphorylation of PDR-1 by PINK-1, leading to its activation. Activated PDR-1 ubiquitinates various proteins on the OMM, which serve as substrates for PINK-1, leading to a feed-forward mechanism and amplifying the mitophagy signal. The poly-ubiquitinated proteins serve as an “eat me” signal that leads to the recruitment of ubiquitin-binding mitophagy receptor SQST-1 (ortholog of SQSTM1 in mammals) or DCT-1 (ortholog of mammalian mitophagy receptor BNIP3L/NIX or BNIP3) [24]. Eventually, the damaged mitochondria are engulfed by the phagophore, or isolation membrane, and form mitophagosomes, which fuse with lysosomes to form mitolysosomes and are ultimately degraded.

Springer et al. identified a single *C. elegans* homolog of human Parkin—*pdr-1* (Parkinson’s disease-related-1) [178]. PDR-1 is a 386-amino-acid protein that shares 28% overall sequence identity and 41% similarity with human Parkin. PDR-1 is ubiquitously expressed in different worm tissues and highly expressed in neurons, where it localizes to both cell bodies and processes. PDR-1 preserves E3 ubiquitin ligase function and acts in a highly conserved ubiquitylation complex. Deletion mutants in *pdr-1(lg103)* make worms more susceptible to proteotoxic stress. Truncated PDR-1(Δaa24–247) in worms results in dysfunctional ubiquitylation machinery, and when the same allele was expressed in cell culture models, it led to toxic aggregates of α-synuclein, the toxic protein implicated in PD [178]. This observation highlights the utility of worm models and their translation into mammalian models of neurodegeneration.

The worm homolog of human PINK1 is *pink-1* [179]. PINK-1 in worms is a Ser/Thr kinase (like human PINK1) containing an N-terminal mitochondria-targeting sequence and shares an overall 36% identity and 54% similarity to its human counterpart. PINK-1 is broadly distributed among different worm tissues and is localized to both the cytoplasmic and mitochondrial compartments. Deletion mutants in *pink-1(tm1779)* lead to a complete loss of function of the protein; however, animals harboring the mutant allele develop normally with a slight reduction in their brood size at 20 °C. These animals are also more susceptible to paraquat (PQ) treatment than wild type. PQ is a bipyridyl herbicide commonly used as a mitochondria-targeted redox cycler to induce oxidative stress, which undergoes enzymatic reduction to produce its cationic radical (PQ+.) and reduces molecular oxygen to generate superoxide radicals [180,181]. An electron microscopy analysis of the mitochondria in *pink-1(tm1779)* worms revealed a 30% reduction in mitochondrial cristae in neurons compared to wild-type worms, and this phenotype was conserved across species [179].

### 4.2. PINK-1/PDR-1-Independent Mitophagy Pathway

Receptor-mediated or ubiquitin-independent mitophagy pathways in mammals involve OMM proteins that contain LC3-interacting region (LIR) motifs and thus can bind to LC3 without the need for ubiquitination or adapter proteins. In mammals, several mitophagy receptors have been identified: BNIP3 (BCL2/adenovirus E1B 19 kDa protein-interacting protein 3), NIX (NIP3-like protein X)/BNIP3L, FUNDC1 (FUN14 domain-containing 1), BCL2L13 (BCL2 like 13), FKBP8 (FK506-binding protein 8), and Prohibitin 2 (PHB2) [182]. Mitophagy mediated by these receptors differs from PINK1/Parkin-mediated mitophagy in that these proteins can establish direct contact between the damaged mitochondria and the growing autophagosome. However, they can also work in concert with PINK1 and Parkin, as suggested above. *C. elegans* orthologs of mammalian mitophagy receptors include DCT-1 (ortholog of mammalian BNIP3L/NIX or BNIP3), FNDC-1 (ortholog of mammalian FUNDC1), and PHB-2 (ortholog of mammalian PHB2).

DCT-1 is an integral protein of the OMM that mediates mitophagy by directly interacting with LGG-1 (ortholog of mammalian LC3) [183]. DCT-1 is widely expressed in different worm tissues, where its expression is upregulated under conditions of low insulin-like signaling by FOXO transcription factor DAF-16. RNAi studies in worms report that DCT-1 deficiency increases mitochondrial content and disrupts the mitochondrial network [165]. DCT-1 also acts in concert with PINK-1 and PDR-1, and DCT-1 overexpression confers resistance to mitophagic stress that is abolished by the depletion of PINK-1 and PDR-1 [165]. Worms with deletion mutants in *dct-1(tm376)*, *pink-1(tm1779)*, and *pdr-1(gk448)* have a plethora of mitochondrial abnormalities, including a depolarized mitochondrial membrane, reduced ATP levels, increased ROS generation, and elevated cytosolic Ca^2+^, but lack changes in mtDNA levels [165,183]. DCT-1 co-localizes with PDR-1 and undergoes ubiquitination, which, however, does not lead to its degradation by the proteasomal pathway [165]. Interestingly, the ubiquitination of DCT-1 is enhanced under oxidative stress conditions, indicating that ubiquitination promotes DCT-1-mediated mitophagy. DCT-1 also plays a role in hypoxia-induced mitophagic response in association with PINK-1/PDR-1 [184]. These observations suggest that DCT-1 acts as the information processing center during mitophagy, which receives both extracellular and intracellular stimuli to mediate the regulation of its downstream effectors.

Our lab identified FNDC-1, the *C. elegans* ortholog of mammalian FUNDC-1 [185,186]. FNDC-1 is an OMM protein required for paternal mitochondria elimination (PME) in worms by programmed mitophagy [185]. In somatic tissue, the loss of function of *fndc-1* reduced mitophagy in response to hypoxic stress in an ATFS-1-dependent manner [186]. ATFS-1 is a transcription factor critical for mediating the mitochondrial unfolded protein response (mito-UPR) [187]. Wei et al. identified and characterized Prohibitin 2 (PHB2 in worms) as an IMM protein priming mitochondria for degradation [188]. PHB2 in mammals works in concert with PINK1/Parkin to mediate mitophagy. The RNAi-mediated knockdown of *phb-2* in worms prevented the degradation of sperm-derived mitochondria in embryos through PME. The discovery of PHB2 unveiled that sequential events at the OMM and IMM are required to clear damaged mitochondria from the cell. These interactions between the different mechanisms of MQC (mitophagy and mito-UPR) to combat stress reflect how these pathways are intertwined and how shifting the delicate balance between these might instigate disease pathology [186]. This concept is discussed in more detail below.

In mammals, cardiolipin and ceramide are lipids containing LIR motifs that play roles in mitophagy [182,189,190]. The externalization of cardiolipin from the IMM (where it is abundant, ~97%) to the OMM (less abundant, ~3%) serves as a cue for apoptosis or mitophagy [191]. Cardiolipin oxidized (CLox) at the OMM serves as a signal for mitophagy. In worms, deletion mutants in the CL synthase gene (*crls-1*) reduced the mitotic proliferation of germ cells and presented mitochondrial abnormalities, depolarized mitochondria, and a reduced number of cristae [192,193]. Another class of lipids involved in mitophagy are ceramides, which contain a sphingosine backbone and fatty acyl chains and are synthesized by ceramide synthases (CerS1–CerS6). Using the mitochondrial toxin Antimycin A, Liu et al. determined the increased co-localization of ceramide and COXIV in body wall muscle cells in *C. elegans*, indicating the involvement of ceramide in mitochondrial health surveillance [193]. However, these studies implicating lipids in mitochondrial health provide indirect evidence of their involvement in mitophagy in worms. More direct studies are needed to understand how lipid-mediated mitophagy is regulated in worms.

## 5. Contribution of the *C. elegans* Model Organism to Better Understand Mitophagy Dysregulation in Alzheimer’s Disease

Mitochondrial defects are uniformly observed in many neurodegenerative diseases. *C. elegans* has long been a premier model for neurobiology and, in recent years, has become well utilized in the study of mitochondrial biology. In 2019, a seminal report by the Bohr and Tavernarakis labs did an excellent job of linking AD-related defects in worms to mitophagy [139]. They showed that the pharmacological stimulation of mitophagy reversed cognitive deficits in worms expressing Aβ or toxic tau in a process that required functional PINK-1 and PDR-1 or DCT-1—results that were recapitulated in APP/PS1 mouse models. Moreover, the associated reduction in the basal oxygen consumption rate (OCR), reduced reserve capacity, increased mitochondrial ROS, and reduced mitophagy (both basal mitophagy and PQ-induced mitophagy) were reversed by mitophagy enhancers. A potentially impactful finding from this study was that tau reduced mitophagy by specifically impairing Parkin recruitment to the damaged mitochondria. These findings in worms parallel those in murine neuroblastoma cells (N2a) transduced with an empty vector (control), htau (2N4R), or P301L mutant tau, highlighting that studies in worm models can be successfully translated to cell-based or animal models [194]. A similar approach demonstrated that spermidine (a naturally occurring polyamine) protected against neurodegeneration in worms that coexpress human Aβ and tau by improving locomotor capacity, restoring associative learning, and upregulating autophagy gene *bec-1*; however, the beneficial effects were lost in *pink-1*, *pdr-1* mutant worms, indicating that a healthy mitophagy pathway is required for spermidine-mediated neuroprotection [195]. Novel steroidal saponins deoxytrillenoside CA (DTCA) and epitrillenoside CA (ETCA) can also stimulate mitophagy and recover neurodegenerative phenotypes in AD worm models, as demonstrated by reduced Aβ aggregation and attenuated Aβ-induced paralysis [196]. Finally, as discussed earlier, our lab showed that the expression of a single copy of phosphomimetic tau (T231E) in the six mechanosensory neurons of *C. elegans* reduced mitophagy in response to oxidative stress and hindered mitolysosomal trafficking, unlike the above studies; baseline mitophagy was not impacted; and wild-type human tau did not elicit an observable phenotype [35]. Further studies indicated that this reduced mitophagy was dependent on the presence of activated *pink-1* in worms but not on the mitochondrial fission regulator *drp-1* [88].

Based on the therapeutic promise of mitophagy inducers, several recent screens have been performed in *C. elegans*. A high-throughput screen of ~45,000 small molecules for the increased accumulation of PINK-1 identified several compounds that delayed paralysis in a *C. elegans* Aβ model in a PINK-1-dependent manner [197]. Using a different approach, Xie et al. combined machine learning with wet lab techniques to select and screen mitophagy inducers from a library of natural products (Macau library) [198]. The 18 AI-selected compounds were tested on both HeLa cells and *C. elegans* to assess mitophagy stimulation. Of the 18, 3 compounds (Quercetin, Kaempferol (Kaem), and Rhapontigenin (Rhap)) successfully stimulated mitophagy in both systems. Kaem and Rhap also improved cognitive deficits in worms expressing human Aβ1-42 and tau (the F3ΔK280 tau fragment and P301L tau). In aggregate, these studies make it apparent that many compounds can stimulate mitophagy. The therapeutic potential of each is likely to be impacted by the precise mechanisms through which this occurs. Since mitophagy is a response to mitochondrial dysfunction, regimens that simply induce dysfunction may be maladaptive overall, albeit useful in an experimental setting. Alternative regimens that stimulate catabolic pathways may have widespread consequences, not all of which are likely to be benign. Ideally, future research would lead to the identification of specific molecular conduits that translate Aβ and tau’s impairment of mitophagy as targets for selective intervention.

## 6. Potential Causes of Dysfunctional MQC in AD

Despite our current appreciation that molecular mediators such as PINK1, Parkin, and BNIP/Nix are likely involved in AD-related mitophagy, the precise mechanism(s) through which MQC is perturbed remains unclear. Molecular genetic studies have suggested associations between MQC-related genes and disease pathology in PD [199], but thus far, this has not been the case in AD, at least for the familial mutations—though PSEN’s ability to regulate mitochondrial calcium in *C. elegans* should be noted [117], as described above. While reconciling the direct and indirect regulation of mitochondrial function in these two diseases is beyond the scope of this review, MQC is widely recognized at the very least to contribute to disease pathology, if not to drive it per se, and hence is positioned as a relevant target for treating AD.

**Figure 3:**
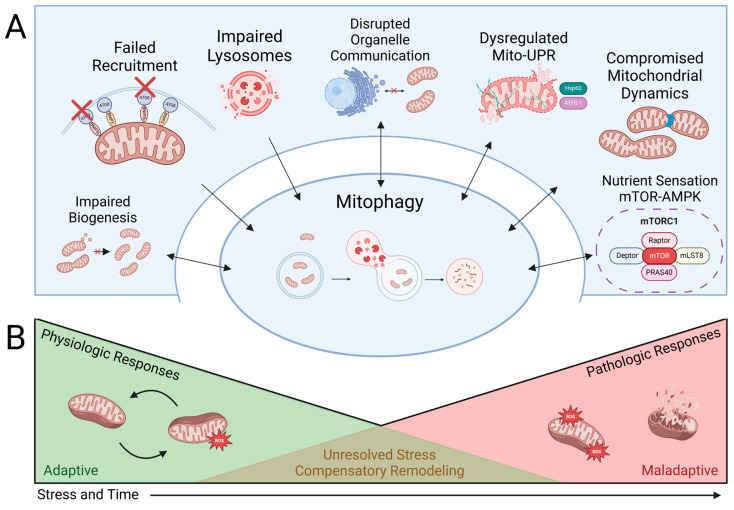
Mitophagy plays a central role in mitochondrial maintenance and homeostasis. Several key stress response pathways feed into mitophagy, which in turn coordinates other maintenance machinery to manage mitochondrial stress (**A**). Acute mitochondrial stress is resolved through the coordinated efforts of these homeostatic pathways, but prolonged stress can cause maladaptive changes (metabolic and epigenetic) which in this model would shift homeostasis toward pathology (**B**). The degree to which the dysregulation of any illustrated MQC pathways contribute to mitochondrial maladaptation and disease pathology in actual AD, as opposed to experimental AD models, is currently uncertain but a topic of great interest. This figure was created using https://www.biorender.com.

Here, we delve into specific phases of mitophagy induction and their regulation, some of which have been connected to Aβ or tau, with the goal of highlighting the different cellular machinery and signaling cascades that can directly, indirectly, independently, or interdependently regulate mitophagy in physiology and pathophysiological conditions like AD (Figure 3). We then circle back to illustrate how the *C. elegans* model has been or could be used to study the selective impairment of these functions.

### 6.1. Failure to Recruit Mitophagy Receptors and Autophagy Receptors Required for Mitophagy

The most direct and potentially selective cause of dysfunctional mitophagy is likely a failure in the recruitment of mitophagy or autophagy receptors observed in neurodegenerative diseases like AD, PD, Huntington’s disease (HD), and Amyotrophic Lateral Sclerosis (ALS), as well as cardiovascular diseases and cancer [200,201,202,203,204]. But how is mitochondrial damage sensing or mitophagy receptor recruitment compromised under diseased conditions? One possibility is the modification or inactivation of mitophagy receptors. PINK1 acts as the first-line damage sensor of mitochondria and can initiate mitophagy. The stress-sensing kinase function of PINK1 corresponds to its mitochondrial import and stabilization on the OMM of damaged mitochondria [205]. Any change in these functions of PINK1 can make damaged mitochondria go unnoticed by clearance machinery. Parkin acts downstream of PINK1, and its recruitment is facilitated by the kinase actions of PINK1 on OMM proteins; therefore, inactive or unrecruited PINK1 on OMM stalls the PINK1/Parkin mitophagy pathway. Moreover, PINK1 acts as a ubiquitin kinase that is essential for the recruitment of autophagy receptors (optineurin, NDP52) for mitophagy. A study in HeLa cells showed that recombinant PINK1 proteins lacking the N-terminal mitochondrial-targeting sequence are cytosolic and fail to recruit OPTN and NDP52 to damaged mitochondria [206]. In addition, Parkin can also be inactivated by chemical modification like S-nitrosylation, which inhibits its E3 ubiquitin ligase activity and the formation of downstream ubiquitin chains on OMM proteins, thereby compromising the generation of “eat me” signals on damaged mitochondria, which are not detected further by autophagy machinery [207]. Further, mitophagy receptors like FUNDC1 and BNIP3 can be activated or deactivated by phosphorylation and dephosphorylation on certain amino acid residues (Ser, Tyr), highlighting another point of control that can be dysregulated in mitophagy [208]. Studies also point out that proteotoxicity by tau can restrict the mitochondrial recruitment of Parkin and confine it in the cytosol [194]. All this information points to the fact that the synchronization of mitochondrial damage sensing with the efficient recruitment of functional mitophagy receptors and the recruitment of autophagy receptors is essential for the removal of damaged mitochondria.

Studies in worms revealed that DCT-1 lies downstream of PINK1/PDR1, indicating that the inactivation of upstream PINK1 can affect the activity of downstream receptors to inactivate mitophagy overall [165]. The supplementation of the NAD+ precursor NMN stimulated DCT-1 co-localization to LGG-1-labeled autophagosomes in nematode neurons, indicating elevated levels of neuronal mitophagy [139]. In a recent study, Chamoli et al. screened a natural product library and discovered a mitophagy-inducing coumarin (MIC) that induced DCT-1-dependent mitophagy and enhanced lifespan in worms in the DAF-12-mediated activation of HLH-30 (transcription factor EB in worms) which is an important regulator of autophagy [209]. The crosstalk between the different mitophagy receptors and the communication axis between mitophagy and autophagy receptors are important targets that should be teased carefully to understand mitophagy dysregulation more accurately.

### 6.2. Compromised Mitochondrial Dynamics

The mitochondrial network is extremely dynamic and varies between cell types, regulating the shape, size, and distribution of mitochondria, reflecting a delicate balance between mitochondrial fission and mitochondrial fusion [210]. An interconnected mitochondrial network can tolerate stress more through the fusion of functional and damaged mitochondria and the sharing of components; however, when damages are beyond a certain threshold, they are eliminated by mitophagy, which requires mitochondrial fission, as does mitochondrial biogenesis. Mitochondrial fusion in worms is regulated by dynamin-related guanine triphosphatases (GTPases) FZO-1 (mitofusin 1/2 in humans) and EAT-3 (Optic Atrophy 1 or Opa-1 in humans), while fission is mediated by DRP-1 (Drp-1 in humans) [211,212]. It is important to note that FZO-1 mediates the fusion of the OMM, whereas EAT-3 mediates the fusion of the IMM [213]. In general, worm loss-of-function mutants are viable, whereas complete knockouts in mice are embryonically lethal [214,215,216]. Among multiple phenotypes, mutants exhibited deficiencies in ATP production and oxygen consumption compared to wild-type animals, which were exacerbated with age [213].

Alterations disrupting the integrity of the fission–fusion pathways can be devastating to mitochondrial function [210]. But how do alternations in mitochondrial dynamics relate to disease pathology in neurons? A simple answer would be that the balance of mitochondrial fission and fusion is extremely important for homeostasis in non-proliferating cells like neurons. It is therefore not surprising that primary mutations in fission and fusion genes cause a variety of neurological disorders (like Autosomal Dominant Optic Atrophy and Charcot–Marie–Tooth Disease) [217,218,219] and have been linked to neurodegeneration in AD, PD, and HD [220,221]. Mitochondria deficient in fission are large entities and have difficulty entering the narrow space of dendrites and axons, causing memory deficits and synaptic dysfunction in neurons [221]. Excessive mitochondrial fission can affect the integrity of cristae and the function of OXPHOS complexes [222]. Interestingly, Twig et al. demonstrated that DRP-1 overexpression reduced mitochondrial mass by 70%, indicating rapid mitophagy events [223]. Parkin overexpression can prevent oxygen glucose deprivation/reperfusion insult in N2a neuroblastoma cells by the degradation of DRP-1, further solidifying connections between mitophagy events and mitochondrial fission [224]. Studies in worms reveal that *fis-1* mutant animals treated with PQ accumulate autophagosomes surrounding mitochondria, indicating that FIS-1-mediated fission is crucial for mitophagy [225].

Several studies have demonstrated that disrupted mitochondrial dynamics also affect mito-UPR signaling. Namely, the knockdown of fission–fusion proteins like *fzo-1*, *eat-3*, and *drp-1* all increase mito-UPR activity [226,227]. Fusion is particularly critical for mitochondrial homeostasis, as it regulates the absorption of fatty acids into mitochondria [228]. When fusion is disrupted, triacylglycerols accumulate heterogeneously within mitochondria and hinder their function, an observation first made in *C. elegans fzo-1* mutants [226]. Interestingly, inducing autophagy suppresses mito-UPR stimulation in these fusion mutants, likely by promoting the beta-oxidation of accumulated triacylglycerols and the subsequent restoration of mitochondrial membrane potential [229]. These studies highlight the interconnectedness of mitochondrial dynamics, mito-UPR, and autophagy.

Studies in AD patients and model systems document the pathological interaction of toxic Aβ and tau with fission regulators in neurons [230]. Aβ promoted DRP1 phosphorylation and increased mitochondrial fission via an Akt-dependent pathway [231]. The Reddy lab has shown that tau can directly interact with DRP1, and a reduction in DRP1 levels improved cognitive behavior, the mRNA and protein levels of mitophagy–autophagy mediators, and mitochondrial biogenesis [232]. As discussed earlier in this review, the pan-neuronal expression of low levels of wild-type human tau in worms can disrupt mitochondrial dynamics as early as the larval stages as indicated by decreased mitochondrial density in the dorsal and ventral cord neurons with smaller and more globular mitochondrial morphology [89]. Therapeutics targeting DRP1 are neuroprotective in AD models [233]. Thus, compromised mitochondrial dynamics can play a role in the downstream stress response pathways like mitophagy.

### 6.3. Impaired Mitochondrial Biogenesis

To maintain a healthy pool of mitochondria within cells, the removal of damaged mitochondria (mitophagy) must be counterbalanced by the synthesis of new mitochondria (mitochondrial biogenesis or mitobiogenesis). Proper coordination between these two interconnected pathways is essential to maintain the quantity and quality of mitochondria in response to an ever-changing metabolic state of cells as well as in response to environmental cues, particularly under diseased conditions. Mitochondrial biogenesis is regulated by mitochondrial transcription factor A (TFAM), nuclear respiratory factors (NRF1 and NRF2), and peroxisome proliferator-activated receptor gamma coactivator 1-alpha (PGC-1α). However, no worm ortholog of PGC-1α has been identified. TFAM is crucial for mitochondrial DNA replication and transcription for mitochondrial biogenesis. TFAM-null mice are embryonically lethal, and TFAM levels directly correlate to the levels of mitochondrial DNA (mtDNA) [234,235]. Reduced TFAM levels result in low mtDNA and a reduced expression of mitochondrial respiratory chain subunits [236]. In neuronal and non-neuronal cells (T-lymphocytes), the genetic deletion of TFAM is associated with mitochondrial and lysosomal dysfunction, reduced endolysosomal trafficking, and autophagy impairment [237]. Therefore, inefficient mitochondrial biogenesis marked by reduced TFAM levels can lead to mitochondrial and lysosomal dysfunction and can be linked to impaired mitophagy as the cell would be inclined to function with damaged mitochondria rather than no mitochondria.

Mitophagy receptors PINK1/Parkin and FUNDC1 also crosstalk with mitochondrial biogenesis pathways. A study on Parkin knockout mice reported a Parkin-interacting substrate (PARIS)-dependent decrease in mitochondrial biogenesis and a PGC-1α-dependent reduction in mitochondrial respiration, confirming Parkin’s role in the regulation of mitochondrial biogenesis [238]. The overexpression of Parkin increased mitochondrial DNA replication and transcription in association with TFAM, whereas the knockdown of Parkin attenuated these processes in proliferating cells [239]. Similarly, the siRNA-mediated knockdown of PINK1 in SH-SY5Y neuroblastoma cells caused decreased mtDNA levels and reduced mitochondrial ATP synthesis, establishing the role of PINK1 in mitochondrial biogenesis [240]. FUNDC1 contains a consensus sequence in its promoter region, facilitating NRF1 binding and regulating mitobiogenesis in a PGC-1α-dependent process [241]. Thus, mitophagy receptors couple mitogenesis and mitophagy, and the crosstalk between these pathways is essential for maintaining healthy mitochondrial and cellular homeostasis, disruptions of which can occur under diseased conditions [242].

Palikaras et al. studied the coupling of mitochondrial biogenesis and mitophagy in *C. elegans* [183]. SKN-1, the *C. elegans* homolog of mammalian NRF2, is a critical regulator of mitochondrial biogenesis and DCT-1-mediated mitophagy. The RNAi-mediated knockdown of *skn-1* produced similar features of mitochondrial dysfunction (mitochondrial morphology defects, membrane depolarization, elevated cytosolic Ca^2+^ levels, and mitophagy impairment) observed with *dct-1* knockdown, and the simultaneous knockdown of *skn-1/dct-1* did not cause a more pronounced mitophagy impairment, indicating that they act in a common genetic pathway regulating mitophagy and mitochondrial biogenesis. In addition, activating *skn-1* can compensate for the reduced basal mitophagy observed in *pink-1/dct-1* mutants [165]. This established a closed feedback loop fact that maintained mitochondrial quality by eliminating damaged mitochondria and synthesizing new mitochondria.

Coming back to AD, studies revealed that AD patients possessed low mtDNA copy numbers and high mutation rates in mtDNA, implying defective mitochondrial biogenesis [236,243]. Sheng et al. reported defective mitochondrial biogenesis in hippocampal neurons from AD patients and cultured M17 cells overexpressing APP with Swedish mutation [244]. Post-mortem studies from the AD brain reported a decreased expression of mitochondrial biogenesis genes PGC1α, TFAM, and NRF2 [245]. The restoration of lysosomal function by NAD+ supplementation improved mitochondrial function in TFAM-deficient cells [237]. NAD+ is often diminished in diseases like AD or as a byproduct of aging [246]. A healthy pool of NAD+ is neuroprotective [247]. Several studies utilizing worm models of AD have linked NAD+ deficiency to mitophagy reduction [40,78,248]. NAD+ supplementation can improve mitochondrial quality in Aβ and tauopathy models in worms by DCT-1- and ULK-1-mediated mitophagy [139].

### 6.4. Impaired Organellar Communication and Trafficking

Mitochondria communicate with other organelles, such as the endoplasmic reticulum, Golgi apparatus, lysosomes, and peroxisomes, through physical contact or shared signaling pathways. The detailed mechanisms through which communication occurs may be species-specific, as mitochondria and vacuoles have been reported to be in physical contact in yeast mediated by Lam6 [249], and more recently, mitochondria–lysosome contact sites have been reported in HeLa cells utilizing correlative light electron microscopy (CLEM) and tethered by the actions of RAB7 [250]. Since mitophagy links mitochondria to lysosomes, impaired mitochondria–lysosome crosstalk is an important aspect to consider for failed mitophagy. Lysosomal storage disorders (LSDs), which result from mutations in lysosomal genes causing a decreased proteolytic activity of lysosomes, are often associated with mitochondrial dysfunction in neurodegeneration and impair mitochondrial turnover by impacting mitophagy [251]. Mutations in PINK1 and Parkin result in defective autophagy–lysosomal machinery and reduced levels of lysosomal protease cathepsin D [252]. Our lab has reported reduced mitolysosome trafficking in *C. elegans* neurons expressing an AD-relevant human tau mutant but not wild-type tau [88]. A recent study by Ryan et al. linked mitochondrial dysfunction in *sel-12* (presenilin) mutants to lysosomal functional impairment [253]. *sel-12*-null mutants have enlarged, alkaline lysosomes arising from dysfunctional inter-organellar communication between mitochondria, the ER, and lysosomes [253]. Using metabolomics, Redmann et al. demonstrated that pharmacological molecules that perturb lysosomal activity (bafilomycin, chloroquine) and inhibit autophagy can result in mitochondrial dysfunction in primary cortical neurons by altering mitochondrial respiration, mitochondrial biogenesis, and the levels of tricarboxylic acid (TCA) cycle intermediates [254]. Furthermore, genetic screens in *C. elegans* have identified autophagic flux as a regulator of mitochondrial stress via metabolite-dependent changes in membrane potential. Manipulations that stimulate autophagy, such as endosomal sorting complex required for transport (ESCRT) disruption, alter metabolite availability, increase mitochondrial membrane potential, and suppress mito-UPR stress signaling—again highlighting the interconnectedness between mitochondrial dynamics, proteostatic stress, and the mito-UPR [226].

Mitochondria–ER contacts are the most well-studied inter-organellar communication. A recent finding in the field of AD is the involvement of the mitochondria-associated endoplasmic reticulum membrane (MAM) in AD pathology [255]. The MAM is a subdomain of the endoplasmic reticulum that communicates with mitochondria. MAMs are rich in lipids like cholesterol and sphingomyelin and facilitate calcium transport, phospholipid synthesis, mitochondrial dynamics, and cholesterol esterification, functions that are perturbed in AD [256,257]. Proteins critical to AD pathology, like presenilins, γ-secretase, and C99 APP, were reported to be enriched in the MAM in AD [255,257]. But how do MAMs impact mitophagy? Recent evidence indicates that mitophagy receptors (PINK1, Parkin, FUNDC1) are components of MAMs, and the recruitment of LC3 to damaged mitochondria occurs at mitochondria–ER contact sites [258]. Using gradient centrifugation, Gelmetti et al. demonstrated an enrichment in PINK1 and Beclin1 in MAMs under basal conditions, which was further increased upon exposure to mitophagic stress (CCCP) [259]. Wu et al. established FUNDC1 as a MAM protein, and the decreased expression of it can affect the formation of mitochondria–ER contact sites, hindering effective organelle crosstalk [260]. Under normoxic conditions, FUNDC1 resides in MAMs, and it is enriched drastically upon exposure to hypoxic conditions where it interacts with the ER protein calnexin [260]. However, contradictory evidence reveals that the disruption of MAMs by disconnecting the mitofusin bridges between mitochondria and ER contact sites prevents mitochondrial dysfunction in *pink-1* or *parkin* mutant flies [261]. MAMs can thus repress mitophagy by precluding specific OMM proteins from activating mitophagy [161].

Because MAMs are a relatively new area of focus, there are only a few reports in worm models on how MAMs may impact mitochondrial physiology. Utilizing transmission electron microscopy (TEM), direct evidence of damaged mitochondria-associated ER membranes was reported in worms exposed to mitochondrial toxins (rotenone/PQ) [262]. Indirect evidence includes a crucial function of MAM microdomains—Ca^2+^ trafficking between mitochondria and the ER and their effects on mitophagy and the mTOR-AMPK axis impacting mitophagy, excellently reviewed elsewhere [263]. Inositol triphosphate receptor (IP3R) mediates Ca^2+^ influx into the mitochondria from the ER, also facilitated by mitochondrial Ca^2+^ uniporters (MCUs). Interestingly, Ryan et al. exhibited that *sel-12* presenilin mutant worms presented reduced autophagosome formation [119]. However, when a *mcu-1*-null mutation was introduced in these worms, it restored autophagic puncta to the levels of wild-type animals, confirming altered mitochondrial calcium levels in autophagic dysregulation. Another report in worms showed that the inhibition of sarco-endoplasmic reticulum calcium ATPase (SERCA) extended lifespan in worms exposed to mitochondrial toxin rotenone, pointing indirectly to the involvement of the MAM in Ca^2+^ dysregulation in mitochondrial health [264,265]. These lines of evidence indicate that mitochondria–ER contacts are important loci for mitochondrial homeostasis and play important roles in mitophagy dysfunction.

### 6.5. Mito-UPR as an Intersecting Stress Signaling Pathway

The ability of cells to upregulate chaperones to manage unfolded mitochondrial proteins was first described in a mammalian cell culture [266], but this phenomenon has since been characterized extensively in *C. elegans* [227]. The mito-UPR in worms is mediated by the transcription factor ATFS-1 [187], the *C. elegans* ortholog of mammalian ATF5 [267]. In general, ATFS-1 is activated by mitochondrial stress, and its targets include genes whose expression will alleviate that stress. The regulation of ATFS-1 utilizes a stereotypical mechanism whereby excluding a transcription factor from the nucleus can facilitate the precise expression of a repertoire of target genes under select conditions where its exclusion is de-suppressed. In ATFS-1’s case, de-suppression is linked to mitochondrial stress through dual intracellular targeting motifs: a mitochondrial targeting sequence (MTS), which allows it to be actively imported into the mitochondria via the TIM and TOM complexes, and a nuclear localization sequence (NLS) which facilitates its import into the nucleus. Under physiological conditions, ATFS-1 is preferentially imported into the mitochondria and is degraded by the protease LON. However, various types of mitochondrial stress—such as excessive ROS-induced damage, an imbalance in mito-nuclear protein ratios, or the presence of unfolded proteins within the mitochondria—can exclude ATFS-1 from TIM-TOM import [187,268]. Moreover, the MTS of ATFS-1 is weak, and thus, even minor perturbations in mitochondrial import efficiency led to mito-UPR activation [269]. When its mitochondrial import is prevented, ATFS-1 instead enters the nucleus where it serves as a bZIP transcription factor and coordinates with DVE-1 and UBL-5 to upregulate heat shock factors and chaperones needed to restore mitochondrial proteostasis, such as *hsp-6*, *hsp-60*, and *clpp-1*, among others [270]. Genes that alter metabolism, general proteostasis, and innate intracellular immunity are also upregulated, indicating that the mito-UPR is a robust signaling axis designed to ameliorate a plethora of mitochondrial stressors [271].

Unsurprisingly, the mito-UPR intersects with mitophagy pathways. In worms, Lim et al. showed that hypoxic sensitivity was coordinated by a functional interaction between the mitophagy receptor FNDC-1 and the central mito-UPR regulator ATFS-1 [186]. In the context of AD, Aβ proteotoxicity in humans, mice, and *C. elegans* has been shown to involve the mito-UPR and mitophagy pathways [272]. However, screens for genetic inducers of the mito-UPR failed to identify mitophagy components [269], while a key regulator of mitochondrial function and the mito-UPR, the SUMO protease ULP-2, altered the expression levels of mitochondrial genes involved in protein import and mtDNA replication but not mitophagy [273]. It is possible that mitophagy and the mito-UPR are not invariably connected under basal conditions but that overt stress unveils a cryptic interaction between these processes. For example, prolonged mito-UPR activation can lead to the erroneous retention of dysfunctional mitochondria. One well-characterized mechanism for this is through the accumulation of mitochondrial heteroplasmy and mutated mitochondrial DNA (mtDNA). Normally, mutations in mtDNA have little effect on the overall health of mitochondrial networks, as hundreds of copies of mtDNA exist, and severely impacted sub-compartments can be selectively cleared via mitophagy. Experiments using mtDNA mutant *C. elegans* demonstrated that ATFS-1 signaling was necessary for mutant mtDNA retention: reducing ATFS-1 activity lowered mutant mtDNA abundance from 60% to 7% [274]. Further studies revealed that ATFS-1 accumulates within mitochondria with mutated mtDNA and can selectively promote their replication through reduced LON activity and increased POLG synthesis [275]. Thus, a chronically active mito-UPR can drastically alter mitochondrial heteroplasmy and homeostasis, which are particularly critical for the health of long-lived, post-mitotic cells like neurons.

Interestingly, mito-UPR activity is high during development, but under normal conditions, its activity decreases with age: a finding which has been independently discovered in *C. elegans*, mice, and humans [276,277]. This decrease in mito-UPR activity is thought to occur through metabolism-dependent chromatin remodeling, a conclusion which is supported by experiments that demonstrated that mito-UPR stimulation does not extend lifespan if performed after L4 [278,279]. Thus, mito-UPR repression may be an evolutionarily conserved mechanism for preventing the drift of mitochondrial homeostasis with age, as described above. Consequently, this may burden other MQC pathways like mitophagy, increasing their relative importance in aging-associated disease. ATFS-1 signaling may also indirectly promote mitochondrial biogenesis by preventing the elimination of mitochondria through mitophagy—a phenomenon that is observed when mito-UPR signaling allows for mutated mtDNA to accumulate—though it is unknown how these two signaling mechanisms coordinate their independent function or whether they are in direct opposition.

While many components of the mito-UPR are conserved between *C. elegans* and mammals, they are not identical. In mammals, the mito-UPR integrates with a larger stress response pathway known as the Integrated Stress Response (ISR). In *C. elegans*, the mito-UPR can function independently of ISR activity; however, ISR activation is an upstream prerequisite for mito-UPR activation in mammals [280]. This incorporates mitochondrial stress into a global stress attenuation program which is mediated through disrupted protein synthesis via the phosphorylation and inhibition of eIF2α [280]. Many genes associated with stress responses contain upstream Open Reading Frames (uORFs), which allow for their selective translation during stressful periods where eIF2α phosphorylation limits protein production [281]. This results in a two-pronged approach to mediate stress: the overall proteostatic burden is reduced by inhibiting global translation, while genes that mediate cell-wide stress resolution selectively increase in abundance. This phenomenon highlights one intersection between protein translation and mito-UPR activation; it is thoroughly reviewed here [271].

Abnormal mito-UPR activation is an emerging theme among neurodegenerative diseases, particularly those tightly associated with mitochondrial dysfunction—like AD and PD. Most neurodegenerative diseases involve aggregation-prone proteins, and proteostasis typically declines with age. Although the deleterious effects of the unfolded protein response in the endoplasmic reticulum (UPR^ER^) [282] have been well characterized, the relationship between the mito-UPR and neurodegeneration has not. The Caldwell lab has demonstrated that a chronic mito-UPR reduces lifespan and limits the clearance of defective mitochondria. Furthermore, mito-UPR activity was synergistically necessary for alpha-synuclein-induced neurodegeneration in a *C. elegans* model [283]. Specific to AD, the mito-UPR was suggested to be more active in post-mortem AD tissues than normal controls. Yet, the transcription factors BAZ2B and EHTM1, which suppress the mito-UPR, have also been shown to exhibit increased translation as AD progresses [284]. Thus, whether mito-UPR induces neuronal dysfunction through chronic activation or whether its inhibition by an upstream event prevents healthy mitochondrial maintenance still needs to be explored in the context of progressive AD pathogenesis.

### 6.6. Impaired Lysosomal Activity

Lysosomes were long thought to be cellular ‘incinerators’, and only recently have their roles in cell metabolism been appreciated [285]. Mitophagosomes formed in early mitophagy must fuse with acidic lysosomes for degradation. Under disease conditions, the elevated pH of the lysosomes can either limit fusion to mitophagosomes or hinder lysosomal proteases [286]. Since lysosomes are the endpoints of autophagy pathways, it is crucial to understand how the autophagic capacity of the cells changes with age and how different tissues respond to different stress stimuli and execute autophagy, especially under diseased conditions. Lysosomes act as the sophisticated signaling hubs receiving information on nutritional state, energetic status, and growth factors and coordinate in regulating the information physically and functionally to “action modules” like mTORC1 pathways that too are tethered on the lysosomal membrane surface [287]. Again, *C. elegans* serve as great models to study lysosomal biogenesis and lysosomal storage disorders (LSDs) [288]. Clokey and Jacobson first observed autofluorescent lipofuscin-like granules in the intestinal cells of *C. elegans* and identified them as secondary lysosomes [289]. Since then, various research groups have investigated lysosomal alterations, autophagy, and aging in worms [290,291,292,293,294,295,296]. Worms possess homologs of all 58 genes identified to date for lysosomal storage disorders, excellently summarized by Voer et al. [297].

Familial AD with autosomal dominant mutations in APP and presenilin often presents with lysosomal dysfunction [125]. AD neurons exhibit an abnormal accumulation of autophagic vacuoles (AVs), which can be the result of lysosomal dysfunction (alkalization) or altered Ca^2+^ homeostasis [298]. A recent study by Zeng et al. in *C. elegans* utilized optogenetic approaches to manipulate lysosomal functions—they developed three optogenetic actuators localized to manipulate different lysosomal functions, namely lyso-NpHR3.0 (lysosomal membrane potential), lyso-ArchT (lysosomal pH), and lyso-ChR2 (hydrolase properties and degradation) [299]. In this study, the light-dependent activation of lyso-ChR2 promoted Aβ clearance and alleviated Aβ-induced paralysis in worms by the activation of the mTOR-mediated autophagy pathway.

Acute mitochondrial stress can trigger lysosomal biogenesis which is dependent on several crucial transcription factors like TFEB and MITF [285]. A very recent study by Wang et al. highlighted another lysosomal regulatory function of TFEB in addition to lysosomal biogenesis. TFEB also plays a critical role in maintaining the acidic pH of lysosomes by regulating lysosomal vacuolar H+ ATPase (vATPase) in a PS19 mouse (expressing P301S mutant tau) model of tauopathy [300]. The nuclear translocation of TFEB and efficient lysosomal biogenesis are dependent on the AMPK-mTOR pathways, the master regulators of metabolism and cell function, which also play key roles in autophagy induction. The mTOR complex assembles on the lysosome surface, and, depending upon amino acid availability, the activation/inactivation of mTOR and its downstream components is crucial for mitophagy. mTOR-AMPK signaling in mitophagy is discussed in detail below.

### 6.7. Signaling Pathways That Control Both Mitophagy and Autophagy—The mTOR-AMPK Axis

The mechanistic target of rapamycin (mTOR), a serine–threonine kinase, is a critical regulator of cellular metabolism that responds to nutrient availability, growth factors, external stress, and more. mTOR complexes are evolutionarily conserved from yeast to mammals and include exclusive mTOR-binding proteins—Raptor (Regulatory Associated Protein of mTOR) and Rictor (Rapamycin-insensitive companion of mTOR) [301,302]. The association of mTOR with Raptor and other regulatory proteins forms the mTORC1 complex, whereas the same with Rictor forms the mTORC2 complex with distinct functions and signaling mechanisms [303,304]. The *C. elegans* orthologs of mTOR, Raptor, and Rictor are LET-363, DAF-15, and RICT-1, respectively [305]. mTORC1 acts as the master regulator of metabolism and acts as the switch controlling the balance between anabolism and catabolism by integrating signals from nutrients, growth factors, and environmental cues, excellently reviewed elsewhere [303,306,307]. From the perspective of this review, we are interested in the functions of mTOR that regulate the autophagy or selective autophagy of mitochondria. A baseline rate of autophagy is essential in all cells to maintain self-renewal functions, particularly in long-lived and highly metabolically active cells like neurons. mTOR carefully regulates autophagy in response to nutrient, oxidative, and bioenergetic stresses, but the mechanisms have only recently become known. A hyperactive mTORC1 is a negative regulator of autophagy–mitophagy and operates through several mechanisms. Firstly, mTORC1 can inhibit transcription factor EB (TFEB, HLH-30 in worms), blocking lysosomal synthesis and inhibiting autophagy [308]. Secondly, mTORC1 can inhibit autophagy by a reduced expression of Unc51 autophagy, activating kinase 1 (ULK1) or locking it in an inactive state by phosphorylation [309,310]. ULK1 is one of the key components in the formation of autophagosomes. The Ser/Thr kinase AMPK critically balances the mTOR–ULK1–autophagy axis. mTORC1 activation inhibits autophagy, while AMPK activation promotes it. AMPK can activate autophagy by directly inhibiting mTORC1 through the phosphorylation of Raptor or indirectly through the phosphorylation of Tuberous Sclerosis Complex 2 (TSC 2) [311]. The AMPK-mediated phosphorylation of ULK1 (S317, S777) also positively regulates autophagy [312,313]. Thus, mTOR-AMPK-ULK1 are like the vertices of a triangle, and the activation or inhibition of each of them directly affects the others in a feedback loop regulating autophagy [314].

Evidence of aberrant mTORC1 hyperactivation has been reported in AD brains and different AD models [315]. An increased phosphorylation of mTOR and its downstream targets ribosomal S6 kinase (p70S6K) and eukaryotic initiation factor 4E (eIF4E) have been reported in the hippocampus and other brain areas of AD patients [315]. Bartolome et al. reported that mTORC1 controls two synergistic processes regulating mitophagy: the initiation of autophagy in general and PINK1/Parkin-mediated mitophagy to target damaged mitochondria to autophagic machinery [316].

Studies in worms utilizing deletion mutants demonstrate that raga-1 (Rag A GTPase ortholog in worms) can regulate mitochondrial dynamics in an unc-64/syntaxin-dependent manner [317]. *sel-12* mutants exhibit hyperactive mTORC1 activity, leading to higher phosphorylation levels of mTORC1 substrate ribosomal S6 kinase. The inhibition of mTORC1 genetically (deletion mutants in *raga-1* and ribosomal S6 kinase *rsks-1*) or pharmacologically (rapamycin) improves proteostasis and the level of autophagosome formation and reverses neurodegeneration observed in *sel-12* mutant worms [119]. Although the role of mTORC2 in regulating autophagy is not well known, deletion mutants in *rict-1* which encodes Rictor, an important activator of mTORC2, can increase DCT-1-dependent mitophagy, and the effect is abolished in the absence of PINK1/PDR1 [318]. This indicates that in addition to mTORC1, mTORC2 may also play roles in mitophagy regulation and requires further investigation. Neural network-based drug repurposing has identified ribavirin as an activator of AMPK and an inhibitor of mTOR (independent of germline loss, caloric restriction, and insulin signaling) in worms [319]. Thus, mTOR activation negatively regulates mitophagy, and inhibiting mTOR proved beneficial in various AD models. More research on the communication axis between mTOR and AMPK and ULK1 would be crucial for the field to design better therapeutic targets in AD.

Nutrient sensing pathways are also key regulators of mitochondrial dynamics. Shpilka et al. demonstrated that the mito-UPR plays a critical role in development, as it links mitochondrial biogenesis to mTORC1 activity. In short, high mTORC1 activity levels during development lead to protein accumulation within mitochondria, which excludes ATFS-1 and promotes mitochondrial expansion [320]. Another study demonstrated that nutrient sensation through Fox0/DAF-16 signaling promotes the accumulation of mutated mtDNA, similarly to chronic ATFS-1 activity [321]. Coincidentally, the accumulation of mtDNA defects was shown to activate mTORC1 and subsequently trigger the mito-UPR in mice [319]. In human hematopoietic stem cells, a regulatory branch of the mito-UPR mediated by SIRT7 and NRF1 and coupled to energy sensation was shown to modulate stem cell quiescence [322,323]. These studies suggest a mechanism through which mito-UPR signaling can become maladaptive: sustained stress can induce a heteroplasmic drift of mtDNA, providing the foundation for chronic mito-UPR activation through cyclical nutrient sensing, mTORC1, and mito-UPR positive feedback. Combined with neurodegenerative mutants that selectively inhibit mitophagy, this highlights a mechanism through which simple mitochondrial perturbations can coalesce into permanent shifts in mitochondrial homeostasis. These shifts may create a cellular context that becomes neurodegenerative with age, when decreased mitochondrial function and impacted proteostasis are common.

## 7. Conclusions

This review attempted to highlight the complexity and interconnectedness of MQC pathways and their relationship to AD in worm models. The field is just beginning to appreciate how mitophagy and mito-UPR signaling interact with other central signaling cascades, such as nutrient sensation through AMPK and mTOR. While energy sensation and mitochondrial maintenance certainly inform one another, more studies are needed to determine the specific mitophagy or mito-UPR machinery that is impacted, whether the effect is direct or indirect, and whether one signaling arm is impacted before the other. Specifically, simply limiting or overactivating mitophagy or mito-UPR signaling may not translate directly to effective therapies. For example, studies have shown that chronically activated mito-UPR signaling can create mitochondrial dysfunction, particularly when combined with proteins associated with neurodegenerative disorders [283]. Furthermore, the integration of the mito-UPR with the ISR in mammalian systems is more complicated than that in *C. elegans*, and it is still being explored, with many downstream transcription factors having unknown functions in mammals. Additionally, resistance to mitophagy stimulation may be a critical early-AD phenotype. Unpublished work from our lab (U. Ganguly, personal communication) suggests that AD-relevant tau PTMs can inhibit mitophagy induction via urolithin A and nicotinamide mononucleotide, indicating that mitophagy suppression may be an inherent part of tau pathology that reduces the efficacy of mitophagy stimulators as a treatment for AD.

One of the caveats to studying mitophagy in AD is that none of the genes regulating mitophagy have been associated with disease pathology. However, basic research in genetic model organisms such as *C. elegans* has helped to develop a more robust understanding of MQC and a nuanced appreciation of how its disruption could contribute to neurodegeneration. While there are undoubtedly limitations in translating work from a model organism to human disease, the uncertainty prevalent in the AD field regarding potentially impactful approaches, much less disease-relevant targets, greatly outweighs these limitations. Case in point, multiple *C. elegans* studies have suggested mito-centric approaches to treating neurodegenerative diseases including AD. To this end, supplying patients with pharmacologic agents that stimulate mitophagy, such as UA, NMN, and NAD+, has been proposed as a potential therapy in the recent literature. Additionally, promoting mitochondrial proteostasis through mito-UPR activation also protects against amyloid-beta-induced toxicity [272], and thus, inducing the mito-UPR may be a promising alternative therapy. In line with this, some studies suggest that a mild impairment of protein translation via pharmacologic agents like cycloheximide may promote health by subtly stimulating the ISR [324]. In conclusion, the identification of novel approaches to treat AD serves as sufficient validation for continued model organism research.

## Figures and Tables

**Table 1:** Examination of mitophagy machinery: tools for studying mitophagy in worms.

Tools Utilized to Assess Mitophagy	References
1. Fluorescence co-localization of mitochondria with autophagosomal markers	
Mitochondria can either be stained by standard mitochondria-specific dyes like tetramethyl rhodamine ethyl ester (TMRE), mitotracker, or rhodamine 6G, or transgenic lines of worms can be generated that express different mitochondrial proteins (localized in OMM, IMM, or matrix) tagged to fluorophores. Autophagosomes can be marked by generating transgenic lines expressing GFP/mCherry-tagged LGG-1 or LGG-2. The co-localization of mitophagic and autophagic markers is indicative of mitophagy. For example, mitochondria-targeted GFP and DsRed-fused LGG-1 were used in this way to assess mitophagy. The limitation of this method is the high rates of false positive results owing to the ability of LGG-1/LGG-2 to aggregate in an autophagy-independent manner.	[163,165,166]
2. Fluorescence quenching	
Mitochondria-targeted Rosella is a fluorescent biosensor consisting of a fusion of pH-insensitive DsRed to pH-sensitive GFP. When mitochondria are engulfed by autophagosomes and fused with lysosomes, the GFP part of the biosensor (sensitive to the acidic interior of lysosomes) is quenched, leaving only the red fluorescence of DsRed. A reduction in the GFP/DsRed fluorescence ratio indicates mitophagy stimulation.	[165]
3. Fluorescent ratiometry: pH	
mKeima is a red-emitting fluorescent protein with a pH-dependent Stokes shift that is excited the most strongly at ~440 nm under neutral conditions and at ~550 nm under acidic conditions. Targeting mKeima to the mitochondrial matrix allows it to be used to monitor mitophagy. Under normal physiological conditions, the pH of the mitochondrial matrix is slightly alkaline (pH 8.0), and 440 nm excitation predominates. Upon the delivery of mitochondria to lysosomes, the environment becomes acidic (pH 4.0), and since mito-mKeima is resistant to acid proteases, the excitation maximum shifts to 550 nm. Moreover, there is also a shift in morphology, with round lysosomes clearly distinguishable from the normal tubular–reticular mitochondria, albeit not from fragmented mitochondria. The ratio of emissions and distinct morphologies has been used to estimate mitophagy in transgenic worms.	[35,167,168]
4. Fluorescent ratiometry: redox	
Another fluorescent probe used to monitor mitophagy is the Timer probe targeted at mitochondria (MitoTimer). Timer is a mutant of the fluorescent protein DsRed (DsRed 1-E5) that fluoresces green (Ex 483 nm, Em 500 nm) when newly synthesized but shifts irreversibly to red (Ex 558 nm, Em 583 nm) when the protein is oxidized (dehydrogenization of Tyr-67). The red/green fluorescence of MitoTimer can be used to monitor mitochondrial dynamics and has been carefully utilized to monitor mitophagy, particularly under circumstances where mitophagy closely matches mitochondrial biogenesis.	[169,170,171]
5. Electron microscopy	
Transmission electron microscopy (TEM) allows us to directly visualize mitochondria surrounded by autophagic (early mitophagy) or lysosomal (late mitophagy) membranes, allowing for an ultrastructural detection of mitophagy. Correlative light and electron microscopy (CLEM) allows us to combine EM and the detection of fluorescent-tagged LC3 proteins (LGG1 and LGG2) in *C. elegans*. However, a major drawback of TEM is the misinterpretation of data due to methodological artifacts that require an expert eye for correct analysis	[172,173,174]
6. Western blotting	
The Western blotting of cell extracts can be used to detect different proteins in the mitochondrial sub-compartments (TOMM20, TIMM23, CYPD, HSP60) and the autophagic proteins required for mitophagy (LC3, p62/SQSTM1). However, the limitation of Western blotting is the inability to monitor mitophagy in different tissues or cell types in worms.	[174]
